# Interactions between wood charcoal and meat types as determining factors of VOC and particle emissions

**DOI:** 10.1007/s10661-026-15221-4

**Published:** 2026-03-24

**Authors:** Alessio Mencarelli, Rosa Greco, Stefano Grigolato

**Affiliations:** Department of Land, Environment, Agriculture and Forestry, University of Padova, Viale Dell’Università 16, 35020 Legnaro, PD Italy

**Keywords:** Cooking fumes, Particulate matter, Volatile organic compounds, Air pollution, Emission factor, Meat composition

## Abstract

Charcoal grilling is a widespread cooking method that contributes significantly to pollutant emissions. This study investigated the release of total volatile organic compounds (TVOCs) and total suspended particulates (TSP) during the grilling of beef steak, chicken breast, and pork belly using lump charcoal and briquettes. Emission factors (EFs) were determined, and the influence of fuel and meat type, meat composition, and operational parameters was assessed through linear mixed-effects models (LMMs). Briquettes produced higher emissions than lump charcoal, with TVOC and TSP about one-third higher. LMMs confirmed that fuel type significantly affects TVOC emissions (*p* = 0.010), while cooking duration (*p* = 0.001), total grilling time (*p* = 0.001), and weight loss (*p* < 0.001) were the main predictors of TSP. Meat type also influenced emissions. Beef steak and chicken breast, despite having a lower fat content, generated significantly more TVOC than pork belly (*p* < 0.05) due to longer cooking times. In contrast, pork belly produced higher TSP through fat dripping. Across both pollutants, weight loss (*p* < 0.001) and the weight loss rate (*p* < 0.001) were robust indicators of pollutant emission. Meat moisture, protein, and ash content were associated with both TVOC and TSP (*p* < 0.001), underscoring the multifactorial origin of grilling emissions. Overall, the results demonstrate that pollutant formation cannot be explained solely by categorical distinctions between fuels or meats. Instead, proximate composition and operational parameters provide the most informative predictors, offering a mechanistic framework for evaluating grilling emissions and supporting strategies to mitigate human exposure.

## Introduction

Grilling is a widely practiced cooking method in both domestic and commercial contexts. Despite the availability of modern cooking technologies such as gas and electric grills, charcoal remains the most commonly used fuel due to the distinctive aroma and flavor it imparts to food (Allais, 2021). Among charcoal-based fuels, two main products dominate the market: lump charcoal, obtained from the pyrolysis of wood, and charcoal briquettes, produced by compacting charcoal dust with binding agents that ensure a uniform shape and more stable combustion (Georgaki et al., 2024; Jelonek et al., 2021; Mencarelli et al., 2025a).

The pleasure and conviviality traditionally associated with grilling come at an environmental and health cost. Whether practiced outdoors or in commercial kitchens, grilling is recognized as one of the most polluting cooking methods. Pollutant formation primarily results from the incomplete combustion of fuel and the pyrolysis of organic matter, particularly fats that drip onto embers or hot surfaces (Xu et al. 2023). These processes generate a complex mixture of air pollutants whose nature and intensity depend on both the fuel used and the characteristics of the cooked food (Liu et al., 2022; Torkmahalleh et al., 2017).

Among the most concerning pollutants are particulate matter (PM) and volatile organic compounds (VOCs). In this context, total suspended particles (TSP) represent a beneficial metric. Unlike size-resolved fractions (PM_1.0_, PM_2.5_, and PM_10_), which capture specific health-relevant dimensions, TSP integrates the overall particle load emitted during grilling. This makes it especially suitable for comparative studies aimed at evaluating how fuels, foods, and cooking practices collectively affect total particulate release (Huang et al., 2016; Mencarelli et al., 2023; Yu et al., 2020).

Total VOC (TVOC) encompasses a wide spectrum of hazardous compounds, including benzene-series hydrocarbons, alkanes, chlorinated and oxygenated VOCs, alkenes, and carbonyls (Liu et al., 2022). Compounds such as BTEX (benzene, toluene, ethylbenzene, and xylene), chloroform, aldehydes (e.g., hexanal, heptanal, and nonanal), styrene, and benzaldehyde are frequently detected in grilling fumes and are classified as carcinogenic or potentially hazardous by international agencies (IARC, 2019; U.S. Epa, 2020; Arı et al., 2020).

These emissions occur in both commercial and domestic settings (Mencarelli et al., 2023). In professional kitchens, grilling has repeatedly been identified as one of the most polluting cooking methods. Cheng et al. (2016) reported that TVOC emissions from grilling were substantially higher than from other Chinese cooking styles, while ElSharkawy and Ibrahim (2022) found that grilling produced the highest pollutant levels among common cooking techniques in Saudi Arabian restaurants. Similarly, Lin et al. (2021) observed that charcoal-based barbecue cooking in Beijing generated PM_2.5_ concentrations four to five times higher than alternative techniques.

In domestic or amateur contexts, similar patterns have been observed. Badyda et al. (2017, 2020) compared charcoal with propane and electric grills and consistently found that charcoal, particularly briquettes, produced the highest pollutant levels. Alves et al. (2022) demonstrated that grilling fish and pork substantially increased both TVOC and particulate emissions compared with charcoal combustion alone, with fat-rich species producing the largest increments. More recently, Xu et al. (2023) characterized emissions from outdoor barbecuing, confirming that both fuel type and food composition markedly affect pollutant levels.

Beyond fuel type, the nature of the grilled food also plays a crucial role. Depending on whether meat, fish, or vegetables are cooked, the composition of the raw food, particularly its fat content, strongly affects emission levels. Higher fat content promotes greater emissions of PM and VOCs as a result of intensified pyrolysis and fat dripping (Alves et al., 2022; Xu et al., 2023). Such emissions pose health risks to both amateur and professional grillers regularly exposed to fumes. Epidemiological and toxicological studies have shown that even short-term exposure can cause eye and respiratory irritation (Lachowicz et al., 2022; Ortiz-Quintero et al., 2022), while chronic exposure, especially in poorly ventilated environments, is associated with elevated risks of respiratory disease and cancer (Badyda et al., 2022).

While these studies have provided valuable evidence, they primarily relied on descriptive comparisons, such as charcoal versus alternative cooking fuels or different types of food, and have not fully disentangled the relative roles of fuel behavior, food composition, and operational parameters in shaping emissions. Consequently, the combined influence of these drivers remains poorly resolved. In a recent study, Mencarelli et al. (2025b) clarified that charcoal properties alone can strongly affect emission concentrations under fuel-only conditions. However, the interaction with food-related factors and cooking dynamics, such as weight loss of both meat and fuel, cooking duration, and meat fat content, has not yet been systematically investigated.

This study addresses this gap by examining the interactions between wood charcoal and meat types as determining factors of VOC and particle emissions. Emission factors for TVOC and TSP were quantified during the grilling of three representative meats, chicken breast, pork belly, and beef steak, using both lump charcoal and briquettes. By integrating variables related to fuel and food performance, including combustion and cooking time, weight loss, and proximate meat composition (moisture, protein, ash, and fat), this work provides a more mechanistic understanding of grilling emissions. Rather than treating fuel and food as separate sources, it explores their combined effect as an integrated system, offering a framework to interpret pollutant formation and guide strategies for exposure reduction.

## Materials and methods

### Meat samples

Beef steak, chicken breast, and pork belly with regular shapes were purchased from a local butcher in Padova, Italy. The beef steak and chicken breast were cut into slices with dimensions of roughly 10 cm × 10 cm × 1.5 cm, while the pork belly slices measured about 5 cm × 10 cm × 1.5 cm, due to the anatomical conformation of this cut, as also reported by Kim et al. (2021). All meat slices were standardized to the same thickness (1.5 cm), which represents the main factor influencing cooking dynamics, thereby ensuring comparability across meat types. The average weight of the beef steak was 127.1 ± 15.1 g, the chicken breast weighed 154.9 ± 13.9 g, and the pork belly weighed 60.3 ± 8.5 g. Three slices from the same meat group (beef steak, chicken breast, or pork belly) were grilled for each grilling test. No marinades or salt were applied to the meat.

Meat samples were stored at 4 °C until use and equilibrated to room temperature immediately before grilling. Proximate composition was determined according to AOAC standard methods: moisture by oven-drying at 105 °C, ash by combustion in a muffle furnace at 550 °C for 16 h, protein by Kjeldahl or Dumas nitrogen determination, and crude fat by ether extraction. Meat weight loss (WL, %) was calculated as follows:

$$\mathrm{WL}\left(\%\right)=\frac{{m}_{\mathrm{RAW}}-{m}_{\mathrm{COOKED}}}{{m}_{\mathrm{RAW}}} \times 100$$

where *m*_RAW_ and *m*_COOKED_ are the weights of the meat slices before and after cooking. The weight loss rate (WLR, g/min) was defined as follows:

$$\mathrm{WLR}\left(\mathrm{g}/\mathrm{min}\right)=\frac{{m}_{{\mathrm{RAW}}^{-m}\mathrm{COOKED}}}{{t}_{\mathrm{COOK}}}$$

where *t*_COOK_ is the cooking time (min).

### Charcoal-based products

Sixteen different types of charcoal were used during the grilling procedure. These included eight samples of lump charcoal and eight samples of charcoal briquettes. The charcoal samples were selected to represent the most common products available on the Italian market and encompassed a wide range of characteristics in terms of geographical origin, wood species, and qualitative properties. Proximate and ultimate compositions are not reported in the present study, as they have already been described in previous works by the same authors (Mencarelli et al., 2025a, 2025b). Charcoal consumption (CC, %) was defined as follows:

$$\mathrm{CC}\left(\%\right)=\frac{{m}_{0}-{m}_{f}}{{m}_{0}}\times 100$$

where *m*_*o*_ and *m*_*f*_ are the weights of the charcoal before starting the grilling test and at the end. The consumption rate (CR, g/min) was calculated as follows:

$$\mathrm{CR}\left(\mathrm{g/min}\right)=\frac{m_0-m_f}{t_\mathrm{total}}$$

where *t*_total_ is the total duration of each grilling test (ignition + cooking time).

### Grilling procedure

The experiments were conducted in an indoor laboratory facility under controlled ambient temperature and humidity conditions to ensure consistent environmental conditions across all tests. Approximately 900 g of lump charcoal or charcoal briquettes were used for each grilling test. The charcoal samples were placed in a garden-style grill (64 cm width × 74 cm depth × 107 cm height). The grilling process was initiated by placing 20 g of BBQ firelighters made of compressed wood chips under the charcoal. An aluminum chimney starter was used for the first 10 min to ensure consistent ignition. After this period, the charcoal was transferred into the barbecue, and the grill grate was positioned.

Ignition time was defined as the interval between the placement of charcoal in the chimney starter and the moment when the grill grate temperature reached 200 °C, measured using a thermocouple connected to a Testo® 175 T3 data logger. At that point, the meat samples were placed on the grill, positioned approximately 10 cm above the fuel bed. Cooking time was defined as the interval between placing the meat slices on the grate and reaching the internal target temperature of 75 ± 2 °C, measured at the geometric center of the slice using a thermocouple connected to the Testo® 175 T3. Total time was calculated as the sum of ignition and cooking times.

Each meat slice was turned only once during grilling, at the midpoint of the cooking process. No marinades or salt were applied to the samples, which had been equilibrated to room temperature before grilling, as described in “Meat samples” section. At the end of each test, both the barbecue and the grilling grate were thoroughly cleaned using neutral detergents, extensively rinsed with water, and completely dried using additive-free absorbent paper, in order to remove combustion residues and food remains and to prevent carry-over effects in subsequent trials.

### Air pollutant sampling

**Fig. 1:**
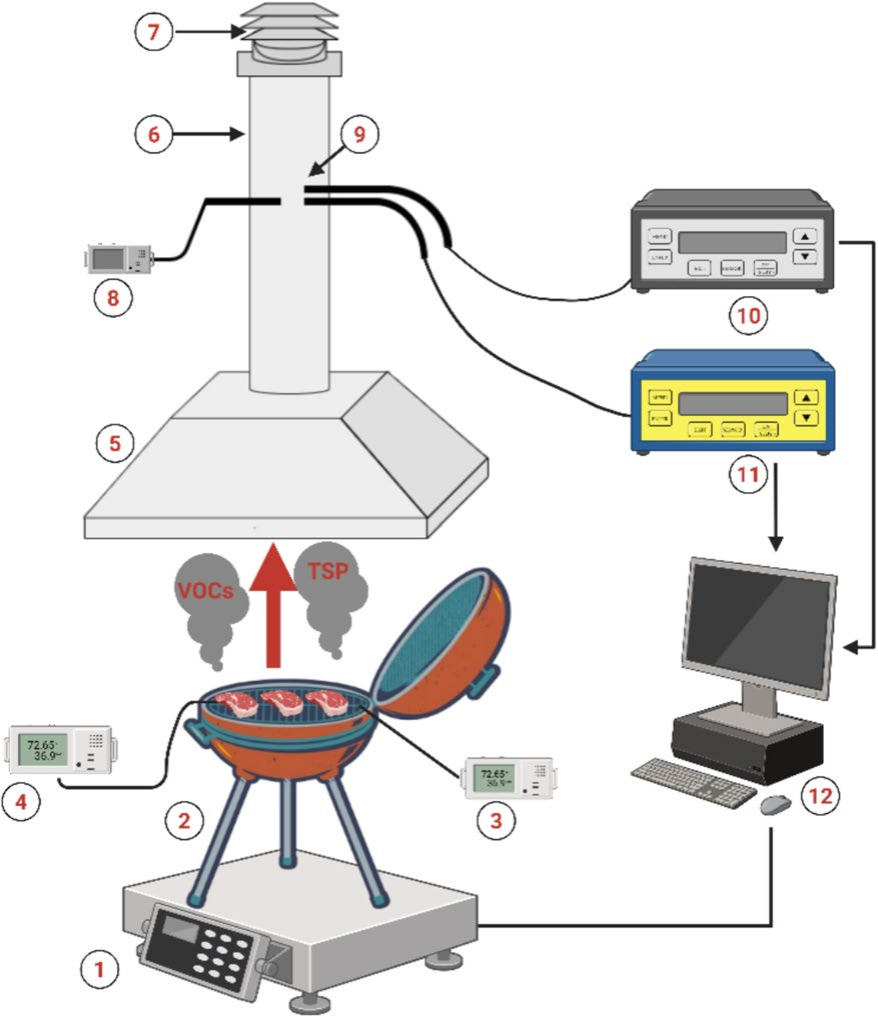
Sampling setup. 1) Digital scale, 2) barbecue, 3) Testo® 175 T3 to measure the grill temperature, 4) Testo® 175 T3 to measure the meat temperature, 5) range hood, 6) exhaust chimney, 7) ventilation fan, 8) anemometer, 9) sampling holes, 10) gas analyzer, 11) particulate matter analyzer, and 12) computer for data registration

Figure 1 illustrates the schematic diagram of the experimental setup for grilling tests using lump charcoal and charcoal briquettes. The barbecue was positioned beneath an aluminum hood to capture fumes and prevent their dispersion. Gas analyzers were installed on the exhaust chimney to measure air pollutant concentrations emitted during charcoal combustion. The barbecue was placed on a digital scale (Radwag® WLC 60/C2/K) to monitor the fuel’s weight loss throughout the grilling process. The exhaust system was set to maintain a fixed air velocity of 2 m/s. Variations in air velocity during the combustion tests were tracked using an anemometer (Delta Ohm® DO2003). Probes for the gas and particulate matter analyzer were positioned at the same height on the exhaust chimney as the anemometer. The sampling point was located 100 cm above the barbecue.

TVOC concentrations were measured using a portable combustion gas analyzer (Sauermann® Si-CA 8500). The total suspended particulate matter (TSP) in the grilling fumes was quantified using the Wöhler® SM 500 analyzer. Both analyzers were factory-calibrated using single-certified calibration gases. To ensure quality control, blank tests were performed not only on charcoal combustion but also on the empty ventilation duct, confirming the absence of background contamination. In addition, the barbecue and hood system was inspected before each trial to verify consistent design and proper sealing of the exhaust path.

Data on gas pollutant concentrations, fuel weight loss, and air velocity were recorded every 30 s throughout the grilling process. Measurements were taken from the ignition of the charcoal product until the grilled meat reached the target temperature. In parallel, a blank analysis of the same duration was conducted to assess emissions resulting solely from charcoal combustion.

The emission factors (EFs) represent the amount of air pollutants released per unit of charcoal burned (g/kg) (Alves et al., 2022). The EFs for the selected air pollutants were calculated using Eq. (1) proposed by Huang et al. (2016) and Yu et al. (2020).

1 $$\mathrm{EF}=\frac{\sum\limits_{i=1}^{n}{C}_{i}{Q}_{i}{t}_{i}}{{m}_{0}-{m}_{f}}$$

Here, *C*_*i*_ is the air pollutant concentration (g/m^3^), *Q*_*i*_ is the sampling flow rate (m^3^/min), *t*_*i*_ is the sampling time of the test (min), *m*_*0*_ is the weight of the charcoal sample before positioning the meat on the grill (kg), and *m*_*f*_ is the weight of the charcoal sample after the grilling (kg).

Ambient temperature and relative humidity were monitored during the experiments but not recorded, since the aim was to ensure stable and comparable experimental conditions rather than to assess environmental variability. Throughout the tests, environmental conditions remained stable, with ambient temperature maintained between 20 and 22 °C and relative humidity ranging from 45 to 55%.

### Statistical analysis

Comparisons among fuels, meats, and operational parameters were initially carried out using one-way ANOVA with Tukey’s HSD test, applied to pollutant concentrations (TVOC and TSP), emission factors (EFs), operational parameters, and meat composition. Before performing ANOVA, the assumptions of normality and homogeneity of variances were assessed using the Shapiro–Wilk test and Levene’s test, respectively. To further evaluate the combined influence of charcoal and meat, emission factors were also analyzed by two-way ANOVA, including “charcoal type” and “meat type” as fixed factors and their interaction, with effect sizes expressed as partial *η*^2^.

In a second step, linear mixed-effects models (LMMs) were applied to identify the role of individual predictors on TVOC and TSP EFs. To minimize collinearity among predictors, models were fitted one effect at a time, with fuel type, meat type, ignition time, cooking time, total time, proximate composition, weight loss, weigh loss rate, charcoal consumption, and consumption rate each tested separately. Sample ID was included as a random intercept to account for heterogeneity among charcoal products. Estimated marginal means and contrasts were used to evaluate categorical predictors, while regression coefficients with 95% Wald confidence intervals were reported for continuous predictors. Model performance was assessed using marginal and conditional *R*^2^ and the corrected Akaike information criterion (AICc).

Statistical significance was denoted as follows: *p* < 0.05 (^*^), *p* < 0.01 (^**^), *p* < 0.001 (^***^), and *p* > 0.05 (n.s.). All analyses were performed using R software (version 4.3.2).

## Results and discussion

### TVOC and TSP concentrations during grilling

**Fig. 2:**
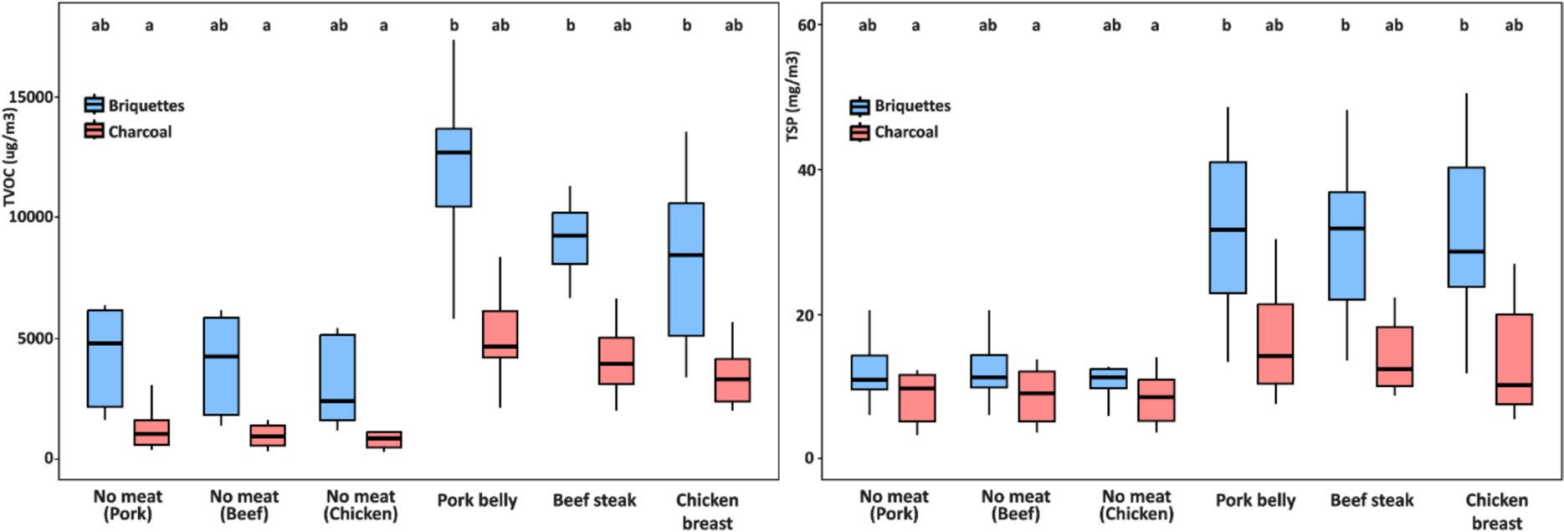
Mean TVOC (A, in µg/m^3^) and TSP (B, in mg/m^3^) concentrations measured during grilling with and without meat, using lump charcoal and briquettes. Different letters indicate statistically significant differences between groups (Dunn’s test, *p* < 0.05)

Time-averaged concentrations of TVOC and TSP measured during grilling tests, with and without meat and using either lump charcoal or briquettes, are reported in Fig. 2. The presence of food markedly increases pollutant emissions compared to fuel-only conditions. Control tests performed with charcoal alone showed substantially lower concentrations (*p* < 0.05), thereby confirming the dominant contribution of food pyrolysis to the formation of air pollutants. This effect was consistent across all meat types, although the magnitude of increase varied depending on the fuel used.

Among fuels, briquettes systematically produced higher pollutant levels than lump charcoal. For VOCs, concentrations ranged from 908 to 11,921 µg/m^3^, with the highest values recorded for pork belly grilled over briquettes. A similar pattern was observed for TSP, where levels ranged from 8.4 to 31.9 mg/m^3^, again with pork belly over briquettes showing the most pronounced increase. When compared to charcoal-only conditions, VOC concentrations increased by up to 324% and TSP by up to 147%. Lump charcoal also led to significant increases relative to controls, but the absolute concentrations were consistently lower than those associated with briquettes.

These findings are consistent with recent literature. Alves et al. (2022) reported that grilling fish and pork substantially increased emissions of CO, CO₂, CH_₄_, HCHO, and VOCs compared with charcoal combustion alone, with the largest increments for fat-rich fish species. Similarly, Xu et al. (2023) highlighted that outdoor barbecuing produces sharp increases in VOC and PM emissions compared to fuel-only tests. Liu et al. (2022) also demonstrated that both food composition and fuel type jointly determine emission levels under controlled conditions.

Overall, these results confirm that the simultaneous contribution of fuel combustion and food cooking strongly amplifies pollutant concentrations in the grilling environment, with briquettes consistently associated with higher emissions than lump charcoal.

### Operational parameters and meat composition

Table 1 presents the operational parameters obtained from grilling meat with charcoal products. Ignition times differed significantly between fuels, with lump charcoal requiring longer times (22–24 min) compared to briquettes (18–19 min) (*p* < 0.05). This indicates a higher ignition efficiency of briquettes, reflected in a shorter ignition time, which can be attributed to the presence of ignition agents and to their higher content of volatile compounds, facilitating the onset of combustion (Huong Huynh et al., 2023; Ju et al., 2020). Briquettes also exhibit a standardized particle size and homogeneous composition, which minimizes variability during ignition. In contrast, lump charcoal consists of irregular fragments often derived from multiple wood species with different densities, resulting in less predictable ignition performance (Jelonek et al., 2020; Mencarelli et al., 2025a). Moreover, briquettes are specifically designed with binders and uniform density to optimize combustion stability (Borowski et al., 2017; Pyshyev et al., 2021).

**Table 1:** Operational parameters of grilling tests with lump charcoal and briquettes across meat types (beef steak, chicken breast, and pork belly)

Variable	Charcoal product	Meat type	Mean + SD
Ignition time (min)	Lump charcoal	Chicken breast	22.4 ± 3.3^a^
		Beef steak	22.1 ± 3.3^a^
		Pork belly	23.6 ± 4.9^a^
	Charcoal briquettes	Chicken breast	18.1 ± 3.4^a^
		Beef steak	19.2 ± 3.6^a^
		Pork belly	18.3 ± 2.0^a^
Cooking time (min)	Lump charcoal	Chicken breast	20.1 ± 4.6^c^
		Beef steak	13.9 ± 3.9^b^
		Pork belly	8.4 ± 1.1^a^
	Charcoal briquettes	Chicken breast	19.6 ± 2.8^c^
		Beef steak	14.4 ± 3.1^b^
		Pork belly	10.3 ± 3.3^a^
Total time (min)	Lump charcoal	Chicken breast	42.4 ± 5.4^b^
		Beef steak	36.1 ± 3.4^a^
		Pork belly	32.0 ± 4.4^a^
	Charcoal briquettes	Chicken breast	37.7 ± 5.9^b^
		Beef steak	33.6 ± 5.2^a,b^
		Pork belly	28.6 ± 3.7^a^
Charcoal consumption (%)	Lump charcoal	Chicken breast	55.9 ± 4.8^a^
		Beef steak	58.9 ± 6.5^a^
		Pork belly	54.4 ± 6.2^a^
	Charcoal briquettes	Chicken breast	55.4 ± 6.0^a^
		Beef steak	55.7 ± 6.1^a^
		Pork belly	48.4 ± 7.6^a^
Consumption rate (g/min)	Lump charcoal	Chicken breast	12.5 ± 1.6^a^
		Beef steak	13.8 ± 1.7^a,b^
		Pork belly	15.3 ± 1.4^b^
	Charcoal briquettes	Chicken breast	12.8 ± 3.2^a,b^
		Beef steak	14.5 ± 3.5^b^
		Pork belly	14.2 ± 4.3^b^

Values are reported as mean ± SD. Different letters indicate significant differences among groups according to Tukey’s post- hoc test (*p* < 0.05)

Cooking time varied significantly among meat types (*p* < 0.05). Chicken breast required the longest duration (20.1 ± 4.6 min), followed by beef steak (13.9–14.4 min) and pork belly (8.4–10.3 min). These differences are attributable to compositional properties: chicken breast, with high water content and low fat, requires prolonged heating to achieve the target internal temperature, while pork belly, with its high fat content (38.7%), reaches cooking temperature more rapidly due to fat rendering and dripping. Beef steak displayed intermediate values, consistent with other reports showing similar trends among these meat categories (Kim et al., 2021). Cooking times did not differ significantly between fuels, indicating that food composition rather than fuel characteristics was the key determinant of this variable.

Total grilling time followed the same pattern, being longest for chicken breast and shortest for pork belly (*p* < 0.05).

Charcoal consumption ranged between 48 and 59% across treatments, with combustion rates between 12 and 15 g/min, and did not differ significantly between fuels or meat types (*p* > 0.05). This indicates that, once ignition is achieved, fuel use is relatively stable, independent of the type of food grilled. This finding is in line with Dias Júnior et al. (2021), who claimed that consumption is primarily influenced by the intrinsic energy properties of the fuel (e.g., fixed carbon, volatile matter, and moisture content), rather than by external cooking conditions or the type of food placed on the grill.

The proximate composition of the meat samples confirmed distinct differences among species (Table 2). Chicken breast and beef steak contained high moisture (71%) and very low fat (1–1.5%), while pork belly had substantially less moisture (42.8%) and markedly higher fat content (38.7%). Protein content was higher in chicken and beef (23%) compared to pork belly (17%). Cooking-induced weight loss was greatest in pork belly (29.7%) and beef steak (28.3%), while chicken breast showed lower values (21.3%). Weight loss rate was highest in beef steak (2.6 g/min), followed by pork belly (2.0 g/min) and chicken breast (1.7 g/min).

**Table 2:** Proximate composition and cooking-related weight loss of beef steak, chicken breast, and pork belly

Meat type	Moisture content (%)	Fat content (%)	Ash content (%)	Protein content (%)	Weight loss (%)	Weight loss rate (g/min)
Chicken breast	71.3 ± 0.8^b^	1.4 ± 0.5^a^	4.2 ± 0.1^c^	23.2 ± 0.8^b^	21.3 ± 4.1^a^	1.7 ± 0.4^a^
Beef steak	71.3 ± 1.0^b^	1.1 ± 0.3^a^	4.0 ± 0.3^b^	23.6 ± 1.2^b^	28.3 ± 5.2^b^	2.6 ± 0.6^b^
Pork belly	42.8 ± 7.9^a^	38.7 ± 10.3^b^	1.3 ± 0.3^a^	17.2 ± 3.2^a^	29.7 ± 5.7^b^	2.0 ± 0.6^a^

Values are reported as mean ± SD. Different letters indicate significant differences among meat types according to Tukey’s post- hoc test (*p* < 0.05)

### EF of air pollutants during meat grilling with lump charcoal and briquettes

#### Influence of fuel type on background emissions

**Fig. 3:**
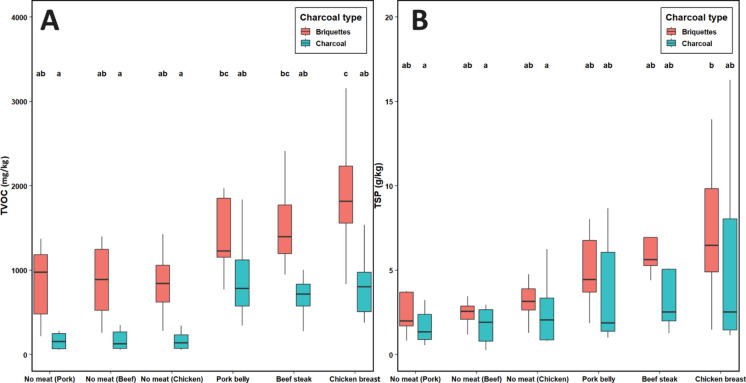
Boxplots of TVOC (**A**) and TSP (**B**) emission factors (EFs) during grilling with lump charcoal and briquettes, with and without meat. Different letters indicate significant differences among groups according to Tukey’s test (*p* < 0.05)

Emission factors (EFs) of TVOC and TSP were strongly influenced by the type of charcoal fuel used (Fig. 3). Under meat-free conditions, no significant differences in TSP EFs were observed between lump charcoal and briquettes (*p* > 0.05), whereas TVOC EFs were significantly higher for briquettes (*p* < 0.05). These differences reflect the combustion properties of the fuels. Lump charcoal consists almost entirely of carbonized wood, while briquettes are produced from a mixture of charcoal dust, binders, ignition agents, and mineral additives (Georgaki et al., 2024; Jelonek et al., 2020). The presence of these additional components promotes incomplete combustion and increases TVOC release. Previous studies have identified petroleum-based ignition agents as contributors to higher gaseous and particulate emissions during charcoal combustion (Campbell & Stockton, 1990; Huang et al., 2016). More recently, Mencarelli et al. (2025b) confirmed that briquettes consistently generate higher TVOC and TSP EFs than lump charcoal across several commercial products.

The composition of briquettes explains these patterns. They generally have higher ash, volatile matter, and moisture content, and lower fixed carbon than lump charcoal (Alzahrani et al., 2024; Dias Júnior et al., 2021). Such properties are positively correlated with increased emission factors of TVOC and fine particles, whereas higher fixed carbon content is negatively correlated with emissions (Mencarelli et al., 2025b; Yu et al., 2020). Furthermore, briquettes are prone to unstable combustion phases, particularly during the early flaming stage, when binders and volatile-rich additives decompose irregularly. This instability results in episodes of incomplete oxidation and consequently higher TVOC and TSP formation (Georgaki et al., 2024; Huang et al., 2016).

#### Effect of meat presence on emissions

The addition of meat during grilling significantly increased both TVOC and TSP EFs compared with fuel-only conditions (*p* < 0.05). Emissions were consistently higher when briquettes were used. These findings are consistent with previous studies that reported marked increases in particulate and TVOC emissions in the presence of meat compared to charcoal combustion alone (Alves et al., 2022; Badyda et al., 2017; Xu et al., 2023). The variability observed among meat types is explained by differences in cooking duration and composition. Despite its higher fat content, pork belly produced lower TVOC EFs than beef steak and chicken breast, which can be attributed to the shorter grilling times of pork belly slices. Chicken and beef required longer exposure to heat to reach the target temperature, thereby enhancing thermal degradation of lipids, proteins, and water, and increasing TVOC release.

The results also showed a high degree of variability within treatments. For briquettes, TVOC coefficients of variation were 24.8% for beef, 44.8% for chicken, and 30.4% for pork, while TSP coefficients of variation were 38.1%, 41.7%, and 40.9%, respectively. For charcoal, TVOC coefficients of variation were 39.8% for beef, 37.1% for chicken, and 38.1% for pork, while TSP coefficients of variation were 38.4%, 60.2%, and 49.7%, respectively. This variability is related both to the heterogeneous composition of briquettes (different types of binders and ignition agents) and to intrasample variability in meat composition, such as the uneven distribution of fat and moisture within cuts. Such heterogeneity has been identified as a critical factor influencing combustion performance and emission stability (Domínguez et al., 2019; Jelonek et al., 2020).

**Fig. 4:**
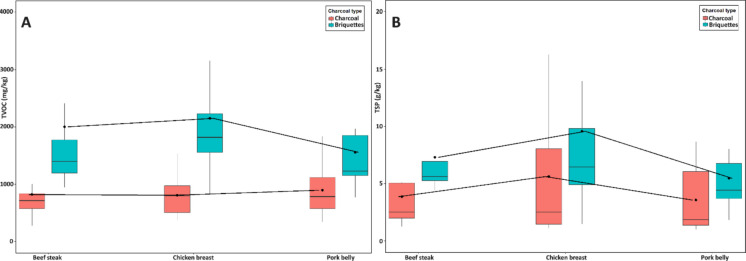
Interaction plots for TVOC (**A**) and TSP (**B**) emission factors as a function of meat type and charcoal type. Points indicate estimated marginal means with 95% confidence intervals

The two-way ANOVA (Table 3) showed a significant main effect of charcoal type on TVOC (*F* = 14.58, *p* < 0.001, partial *η*^2^ = 0.258) and a near-significant effect on TSP (*F* = 3.96, *p* = 0.053, partial *η*^2^ = 0.086), whereas meat type had no effect on either response (all *p* ≥ 0.27) and the charcoal × meat interaction was not significant (*p* > 0.05). In line with these outcomes, the interaction plots (Fig. 4) display approximately parallel trends across meat types, indicating additive rather than synergistic effects. Consistently, the highest emission scenarios were observed with briquettes regardless of meat type.

**Table 3:** Two-way ANOVA results for emission factors (EFs) of TVOC and TSP as a function of charcoal type, meat type, and their interaction

Response	Factor	df	*F*-value	*p*-value	Partial *η*^2^
TVOC	Charcoal type	1	14.58	< 0.001^***^	0.258
	Meat type	2	0.30	0.745^n.s^	0.014
	Charcoal x meat	2	0.55	0.582^n.s^	0.025
TSP	Charcoal type	1	3.96	0.053^n.s^	0.086
	Meat type	2	1.35	0.271^n.s^	0.060
	Charcoal x meat	2	0.16	0.854^n.s^	0.007

Partial η2 values indicate effect size. Statistical significance is reported as: *** p < 0.001, ** p < 0.01, * p < 0.05; n.s. = not significant

The increase in emissions with meat presence reflects a dual mechanism: (i) the effect of fuel combustion, particularly relevant for briquettes, and (ii) the pyrolysis of food constituents. The latter includes lipid oxidation and dripping, protein degradation, and Maillard reactions, which together release a complex mixture of organic compounds and contribute to particulate formation (Duedahl-Olesen & Ionas, 2022; Lee et al., 2016). Fat dripping can also trigger secondary combustion on hot embers, releasing condensable organic compounds and fine particles, as demonstrated in experiments where oil was dripped onto burning charcoal (Yu et al., 2020).

These combined mechanisms highlight the complexity of emission processes during grilling, where both the fuel matrix and the properties of the food jointly contribute to pollutant release. While the ANOVA identified differences attributable to fuel type (but not meat type), it does not reveal which variables are the main drivers of TVOC and TSP emissions. To address this, linear mixed-effects models were applied, offering a quantitative framework to assess the relative importance of compositional and operational predictors.

### Impact of factors affecting EF during grilling

**Fig. 5:**
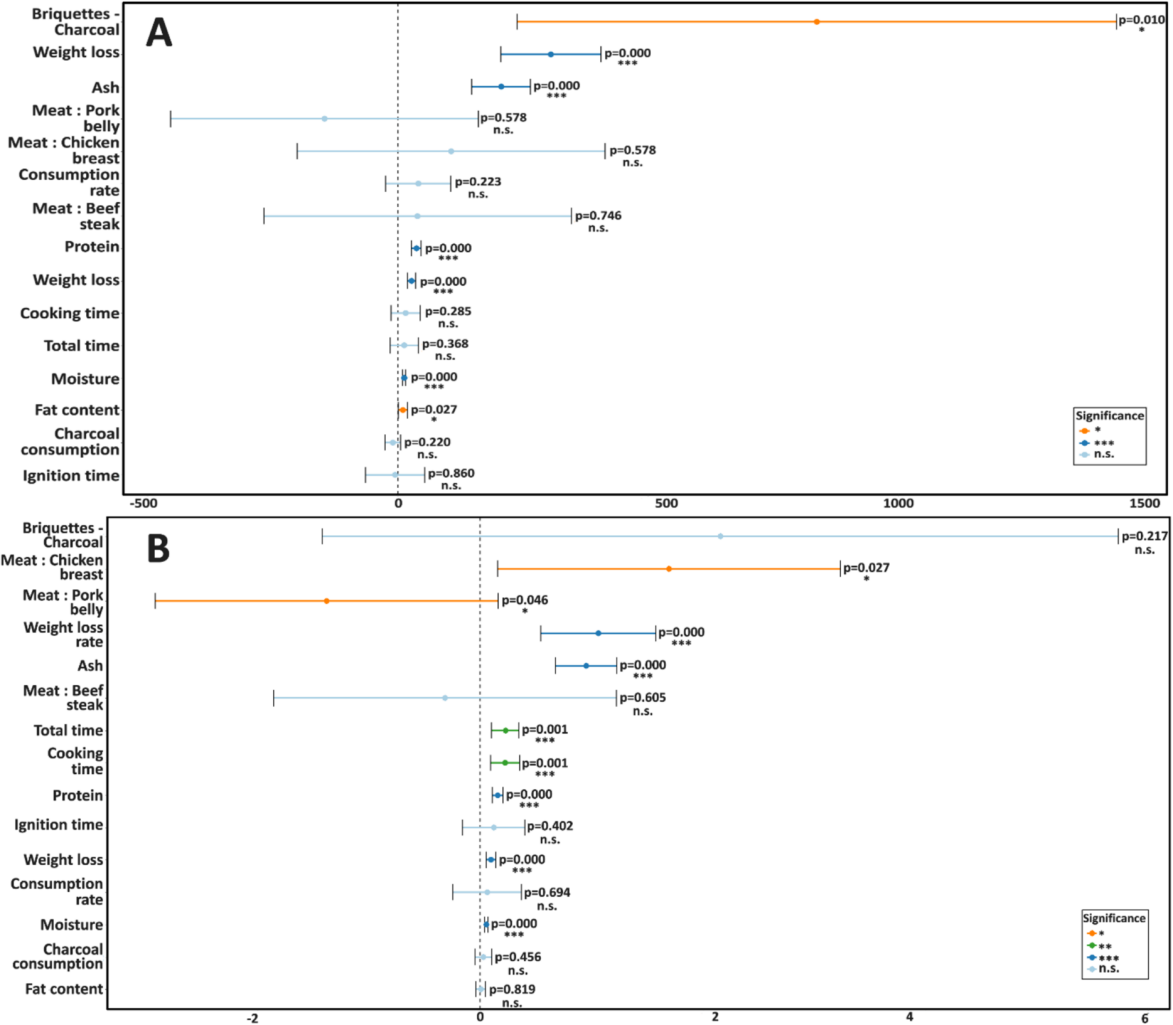
Forest plots of the linear mixed-effects models for (**A**) TVOC and (**B**) TSP emission factors. Points represent regression coefficients (*β*) and horizontal lines indicate 95% confidence intervals. Colors denote statistical significance (^***^p < 0.001, ^**^p < 0.01, *p < 0.05, n.s. = not significant)

The linear mixed-effects models (LMMs) clarified the contributions of fuel type, meat composition, and operational parameters to the emission factors (EFs) of TVOC and TSP (Table 4). In line with the descriptive patterns (Fig. 3 and 4), the models quantified which variables significantly predicted pollutant formation and revealed distinct drivers for TVOC and TSP (Fig. 5). Model performance was satisfactory, with marginal R^2^ values reaching 0.20 for TVOC and 0.15 for TSP, and conditional *R*^2^ values up to 0.66 and 0.52, respectively, indicating that both fixed and random effects contributed to the explained variance.

**Table 4:** Results of linear mixed-effects models (LMMs) testing the influence of fuel type, meat type, operational parameters, and proximate composition on emission factors (EFs) of TVOC and TSP. Reported are regression coefficients (*β*), standard errors (SE), 95% confidence intervals (CI), p-values, marginal and conditional *R*^2^, and corrected Akaike information criterion (AICc)

Response	Effect	Level	*β*	SE	CI (low)	CI (high)	*p*-value	*R*^2^ marg	*R*^2^ cond	AICc
TVOC	Charcoal product	Briquettes—charcoal	837.1	279.2	238.4	1435.9	0.010^*^	0.20	0.47	1529.8
	Meat type	Beef steak	39.7	121.2	−267.7	347.0	0.746^n.s^	0.01	0.71	753.6
		Chicken breast	106.7	121.2	−200.6	414.1	0.578^n.s^	0.01	0.71	753.6
		Pork belly	146.4	121.2	−453.8	161.0	0.578^n.s^	0.01	0.71	753.6
	Time	Ignition time	−5.3	30.0	−64.1	53.4	0.860^n.s^	0.00	0.46	1541.6
		Cooking time	16.0	14.8	−13.1	45.0	0.285^n.s^	0.01	0.44	1541.9
		Total time	12.9	14.2	−15.0	40.7	0.368^n.s^	0.01	0.46	1542.3
	Weight loss	Weight loss	27.3	4.1	19.4	35.3	0.000^***^	0.17	0.66	1509.5
		Weight loss rate	306.1	51.2	205.8	406.3	0.000^***^	0.13	0.66	1511.4
	Proximate composition	Fat content	10.4	4.6	1.3	19.5	0.027^*^	0.03	0.49	1540.4
		Moisture content	12.5	1.6	9.3	15.8	0.000^***^	0.19	0.69	1503.3
		Protein content	37.4	4.8	28.0	46.9	0.000^***^	0.19	0.70	1500.3
		Ash content	206.3	30.0	147.5	265.1	0.000^***^	0.17	0.66	1504.2
	Charcoal consumption	Charcoal consumption	−9.9	8.0	−25.6	5.8	0.220^n.s^	0.02	0.40	1543.1
		Consumption Rate	40.8	33.3	−24.5	106.1	0.223^n.s^	0.02	0.55	1540.3
TSP	Charcoal product	Briquettes—Charcoal	2.2	1.7	−1.4	5.7	0.217^n.s^	0.06	0.51	528.0
	Meat type	Beef steak	−0.3	0.6	−1.9	1.2	0.605^n.s^	0.05	0.72	276.9
		Chicken breast	1.7	0.6	0.2	3.2	0.027^*^	0.05	0.72	276.9
		Pork belly	−1.4	0.6	−2.9	0.2	0.046^*^	0.05	0.72	276.9
	Time	Ignition time	0.1	0.1	−0.2	0.4	0.402^n.s^	0.01	0.51	533.9
		Cooking time	0.2	0.1	0.1	0.4	0.001^***^	0.08	0.46	525.9
		Total time	0.2	0.1	0.1	0.3	0.001^***^	0.10	0.52	524.3
	Weight loss	Weight loss	0.1	0.0	0.1	0.1	0.000^***^	0.09	0.60	519.4
		Weight loss rate	1.1	0.3	0.5	1.6	0.000^***^	0.07	0.60	518.7
	Proximate composition	Fat content	0.0	0.0	0.0	0.0	0.819^n.s^	0.00	0.49	538.3
		Moisture content	0.1	0.0	0.0	0.1	0.000^***^	0.16	0.68	504.2
		Protein content	0.2	0.0	0.1	0.2	0.000^***^	0.15	0.66	504.8
		Ash content	1.0	0.1	0.7	1.2	0.000^***^	0.15	0.68	498.5
	Charcoal consumption	Charcoal consumption	0.0	0.0	0.0	0.1	0.456^n.s^	0.01	0.53	536.8
		Consumption Rate	0.1	0.2	−0.2	0.4	0.694^n.s^	0.00	0.52	534.3

*** p < 0.001, ** p < 0.01, and * p < 0.05; n.s. = not significant

For VOCs, fuel type was the dominant predictor, with briquettes producing on average 837 ± 279 mg/kg more emissions than lump charcoal (*p* = 0.010, AICc = 1529.8). This confirms the descriptive evidence and highlights the central role of combustion efficiency, reinforcing that the lower fixed carbon and higher volatile and ash contents of briquettes strongly promote incomplete oxidation and VOC release (Georgaki et al., 2024; Jelonek et al., 2020; Mencarelli et al., 2025b). Several food-related predictors were also significant. Moisture (*β* = 12.5 ± 1.6, *p* < 0.001), protein (*β* = 37.4 ± 4.8, *p* < 0.001), and ash content (*β* = 206.3 ± 30.0, *p* < 0.001) were all positively associated with TVOC emissions, indicating that the pyrolysis of water-rich and protein-rich tissues contributes substantially to volatile formation. The positive association with ash content may reflect the catalytic role of minerals in thermal degradation reactions (Liu et al., 2022; Pikmann et al., 2024). Fat content, although significant (*β* = 10.4 ± 4.6, *p* = 0.027), explained less variability than expected, which suggests that lean meats such as beef and chicken may generate high TVOC levels through longer cooking times and enhanced protein degradation (Kim et al., 2021; Lee et al., 2016). Strong positive coefficients for weight loss (*β* = 27.3 ± 4.1, *p *< 0.001) and weight loss rate (*β* = 306.1 ± 51.2, *p* < 0.001) further support that samples losing more mass during cooking, through evaporation, fat rendering, and structural breakdown, systematically released higher amounts of TVOC.

For TSP, a different pattern was observed. Cooking time (*β* = 0.2 ± 0.1, *p* = 0.001) and total grilling time (*β* = 0.2 ± 0.1, *p* = 0.001) were significant predictors, showing that prolonged exposure sustains the release of combustion residues and condensable organics. Weight loss (*β* = 0.1 ± 0.0, *p *< 0.001) and weight loss rate (*β* = 1.1 ± 0.3, *p* < 0.001) also emerged as strong positive predictors, reinforcing the role of dehydration and dripping as key drivers of particulate emissions. Proximate composition further contributed, with moisture (*β* = 0.1 ± 0.0, *p* < 0.001), protein (*β* = 0.2 ± 0.0, *p* < 0.001), and ash content (*β* = 1.0 ± 0.2, *p* < 0.001) all positively associated with TSP, confirming that particle formation is a multifactorial process involving both lipid and protein pyrolysis (Alves et al., 2022; Xu et al., 2023). Although ignition time (*β* = 0.1 ± 0.1, *p* = 0.402) was not among the strongest predictors in this dataset, its inclusion in the model is noteworthy, as it likely reflects emissions from the initial flaming stage of charcoal combustion, when incomplete oxidation dominates and particulate emissions peak (Huang et al., 2016; Vicente et al., 2018). Interestingly, categorical meat type was less informative than proximate composition. Chicken breast was positively associated with TSP (*β* = 1.7 ± 0.6, *p* = 0.027), while pork belly showed a negative coefficient (*β* = −1.4 ± 0.6, *p* = 0.046) despite its higher fat content. This apparent contradiction can be explained by the fact that dripping fat and secondary combustion on embers, while recognized as important mechanisms of particulate release (Alves et al., 2022; Xu et al., 2023), may not consistently outweigh the effects of longer cooking times and higher protein content in leaner meats. The role of intrasample variability, such as uneven distribution of fat and water within cuts, likely contributes to this complexity (Domínguez et al., 2019).

Overall, the LMMs revealed partially overlapping but distinct mechanisms. TVOC emissions were mainly driven by fuel-related combustion efficiency and the extent of food mass loss, whereas TSP emissions were more strongly tied to cooking duration and proximate composition. The repeated association of weight loss with both pollutants underscores its role as a reliable integrative indicator of emission intensity. Compared with previous works that primarily contrasted fuels or food categories (Alves et al., 2022; Badyda et al., 2020; Mencarelli et al., 2025b; Xu et al., 2023), the present models provide quantitative evidence for the role of compositional and operational parameters in shaping grilling emissions, and emphasize that weight-loss metrics are stronger predictors than categorical descriptors such as meat type.

### Limitations and future perspectives

This study has some limitations that should be acknowledged. First, particulate matter was analyzed as total suspended particles (TSP) without discriminating between size fractions (PM_1.0_, PM_2.5_, and PM_10_), which are known to have different health implications. Nevertheless, TSP was selected as an integrative indicator of overall emissions, which adequately supports the comparative objectives of this study. Future investigations focusing on particle size distributions could provide a more detailed understanding of health-relevant fractions and allow direct comparison with air quality standards.

Second, while the study measured TVOC, no chemical speciation was performed. This approach was justified by the aim of identifying general emission trends across fuels and meats, rather than characterizing individual compounds. However, compound-specific analyses, for example, through GC–MS, would provide deeper insight into the formation pathways of hazardous pollutants and could support more refined exposure and risk assessments.

Despite these limitations, the study provides a robust and original contribution to the understanding of grilling emissions. By highlighting the role of fuel type, operational parameters, and proximate composition, it establishes a solid foundation for future work aimed at refining chemical characterization, assessing size-resolved particles, and evaluating emissions under realistic outdoor conditions. Such developments will strengthen the relevance of laboratory findings and support practical strategies to mitigate human exposure.

## Conclusions

This study provides new insight into the mechanisms driving pollutant emissions from charcoal grilling, emphasizing the interaction between fuel behavior and meat properties. The results indicate that pollutant formation arises from interconnected processes rather than from the independent influence of fuel or food.

Charcoal briquettes consistently produced higher TVOC emissions than lump charcoal, confirming the influence of fuel composition and combustion efficiency on the release of volatile compounds. In contrast, TSP emissions were more strongly determined by cooking duration and meat composition, highlighting the role of food-related processes such as fat dripping, dehydration, and surface charring.

Across both pollutants, total and relative weight loss emerged as reliable indicators of emission intensity, reflecting the combined effects of moisture evaporation, fat volatilization, and structural degradation of the meat. Compositional variables such as moisture, protein, and ash, together with operational parameters including grilling time, explained emission variability more effectively than categorical factors such as fuel or meat type.

Overall, grilling emissions cannot be attributed to fuel or food categories alone. They are shaped by the interaction between the physical dynamics of combustion and the compositional characteristics of the food, which together define the thermal and reactive environment of cooking. Recognizing this interaction provides a coherent framework for interpreting emission variability across different grilling conditions. It supports the development of strategies to reduce human exposure while maintaining the sensory value of charcoal-based cooking.

Future research should extend this approach to compound-specific VOC profiling and size-resolved particle characterization, as well as to field measurements under real-world cooking scenarios. Such efforts will enhance the mechanistic understanding of grilling emissions and contribute to the development of effective mitigation strategies that minimize health risks while preserving the cultural and sensory appeal of charcoal grilling.

## Data Availability

The datasets generated and analyzed during the current study are available in the Research Data Unipd repository at https://researchdata.cab.unipd.it/1595, with the identifier 10.25430/researchdata.cab.unipd.it.00001595.
